# Hypoxia‐Inducible Factor 1α in the Aortic Wall: Protective Adaptation or Marker of a Vulnerable Phenotype?

**DOI:** 10.1096/fj.202603791R

**Published:** 2026-09-11

**Authors:** Nimrat Grewal

**Affiliations:** ^1^ Department of Cardiothoracic Surgery Amsterdam University Medical Center location AMC Amsterdam the Netherlands; ^2^ Aortic Institute at Yale‐New Haven Hospital Yale University School of Medicine New Haven Connecticut USA

Zhao et al. present a rigorous and mechanistically comprehensive study demonstrating that the prolyl hydroxylase inhibitor roxadustat (ROX) attenuates β‐aminopropionitrile (BAPN)‐induced thoracic aortic dissection (TAD) in mice by stabilizing hypoxia‐inducible factor 1α (HIF‐1α), thereby activating PINK1/Parkin‐mediated mitophagy to suppress vascular smooth muscle cell (VSMC) ferroptosis [1]. The breadth of the approach, spanning in vivo survival, imaging, mitochondrial respirometry, and genetic silencing of both HIF‐1α and PINK1, is commendable, and the repositioning of a clinically approved agent toward aortic disease is an attractive translational prospect.

However, interpreting HIF‐1α as a uniformly protective, adaptive factor whose pharmacological amplification is desirable requires caution, not because of any limitation in experimental rigor, but because observations in human aortic tissue point to a more context‐dependent, and at times opposite, role. HIF‐1α is indeed elevated in human thoracic aortic dissection tissue, as the authors themselves note, consistent with its established upregulation in human aortic aneurysm disease [2]; yet elevated expression in diseased tissue does not, in itself, establish a protective function.

## 1 In the Human Aortic Wall, HIF‐1α Marks Vulnerability Rather Than Protection

In a study of bicuspid (BAV) and tricuspid aortic valve patients, with and without dilatation, we found HIF‐1α, together with phosphorylated c‐Kit (pc‐Kit), to be a marker of cellular dedifferentiation that characterized the aortic wall susceptible to future aortopathy [3]. Non‐dilated BAV aortas segregated into two immunohistochemically distinct subgroups: one expressing high pc‐Kit, HIF‐1α, eNOS, and MMP9, closely resembling already‐dilated BAV walls, and another essentially devoid of these markers. In the human aorta, HIF‐1α thus marked the vulnerable, remodeling‐prone phenotype rather than a protective one, consistent with the maturation defect and delayed smooth muscle cell differentiation that characterize the BAV aortic wall [4].

## 2 In the Same Acute Dissection Setting, Human Evidence Links HIF‐1α to Injury Rather Than Protection

This context dependence is most striking within the very setting the authors model. In human acute Stanford type A aortic dissection, oxidative stress has been shown to drive vascular smooth muscle cell damage through an HIF‐1α/HO‐1 mediated ferroptosis axis [5]. In the same acute dissecting aorta, then, HIF‐1α has been placed on the injurious rather than the protective side of the ferroptotic pathway. That two independent lines of evidence assign HIF‐1α opposite roles in comparable tissue argues that its net effect is governed by cell state, disease context, and the route of its activation, rather than being uniformly cytoprotective.

## 3 The Upstream Route to HIF‐1α Differs and May Dictate Its Downstream Role

Zhao et al. stabilize HIF‐1α through the canonical PHD/hypoxia pathway. In human specimens, HIF‐1α instead appeared downstream of MMP9‐mediated release of stem cell factor and consequent c‐Kit phosphorylation [3], a hypoxia‐independent, remodeling‐associated route, with HIF‐1α localizing atypically to the cell membrane. The same transcription factor, reached through different upstream mechanisms, need not exert the same downstream effect.

## 4 Disease Context Matters: Acute Dissection Versus Chronic Dilatation

BAPN models acute elastolytic dissection, whereas BAV aortopathy is a chronic dilatory process. Notably, a recent detailed characterization of this model shows that dissection predominates in the descending thoracic aorta, corresponding to Stanford type B, whereas luminal dilatation is more pronounced in the ascending aorta [6]. This regional distinction is itself relevant to translation, because bicuspid aortopathy is predominantly an ascending and aortic root disease, so the segment in which the murine phenotype is most overtly dissecting is not the segment most affected in bicuspid aortic valve disease. If, in a subset of human aortas, HIF‐1α marks or participates in a dedifferentiated, remodeling‐prone media, chronic pharmacological HIF‐1α stabilization is not self‐evidently benign. This adds a wall‐intrinsic consideration to the systemic cautions the authors already acknowledge (VEGF‐mediated angiogenesis and oncogenic potential) and argues that translation of ROX to human aortic disease should be preceded by patient stratification, for example by VSMC differentiation markers or the pc‐Kit/HIF‐1α susceptibility signature, rather than by an assumption of uniform benefit.

## 5 Excessive or Chronic Mitophagy May Itself Be Maladaptive in the Diseased Aortic Wall

A further layer of context concerns the difference between prevention and treatment. Murine studies typically apply short‐term prevention, whereas patients receive lifelong treatment of established disease, in which the aortic wall already harbors a dedifferentiated smooth muscle cell phenotype and preexisting mitochondrial dysfunction. In this setting, enhanced or chronic mitophagy is not unambiguously protective, because excessive clearance of mitochondria may deplete the limited pool of functional organelles needed to meet cellular energy demand, aggravating rather than relieving injury. Such maladaptive mitophagy has been described in heart failure, where enhanced Parkin‐mediated mitophagy and mitochondrial fragmentation contributed to disease severity [7, 8]. The history of doxycycline in aortic aneurysm is instructive. Widely regarded as protective in short‐term murine prevention studies, it advanced aortic aneurysm disease in patients on chronic treatment [9] and did not influence already‐established aneurysms in mice [10]. Because doxycycline also inhibits mitochondrial activity, it may harm cells already burdened by mitochondrial damage while sparing healthy ones, illustrating that diseased and healthy vascular smooth muscle cells, and prevention versus treatment, are not equivalent. The therapeutic consequences of amplifying HIF‐1α‐driven mitophagy may therefore differ markedly between the preventive murine setting and the chronically diseased human aorta.

## 6 Path Forward: Stratify by Vascular Smooth Muscle Cell Phenotype

These human observations are, importantly, associative and immunohistochemical, whereas Zhao et al. manipulate HIF‐1α causally, through genetic silencing and pharmacological inhibition. A marker of vulnerability in the human wall and a protective mechanism in the murine wall cannot be equated, and the causal role of HIF‐1α in human aortic disease therefore remains genuinely unresolved. The protective effect described here is attributed to VSMC mitophagy without stratification by VSMC phenotype, yet differentiation state was precisely what distinguished susceptible from resistant human aortas in our cohort [3]. Although the authors validate their findings in a human aortic smooth muscle cell line, that model does not capture the phenotypic heterogeneity of the intact aortic wall. Validation in patient‐derived human aortic tissue, stratified by VSMC differentiation status and the pc‐Kit/HIF‐1α susceptibility signature, would therefore meaningfully strengthen the translational case, alongside confirmation in female animals, which the authors themselves note as a limitation. An important caveat is that surgically obtained human aortic tissue is typically harvested at an advanced disease stage and may capture downstream consequences rather than the mechanisms that initiate disease, so such analyses should ideally be complemented by earlier‐stage or longitudinally sampled tissue and by models that capture disease initiation.

## 7 Conclusion

This is a rigorous and thought‐provoking study that convincingly establishes a protective HIF‐1α/mitophagy axis in the murine dissecting aorta. Reconciling that finding with the association of HIF‐1α with vulnerability in the human dilating aorta remains, in my view, the pivotal question determining whether HIF‐1α stabilization can be safely translated to patients. Given the complex pathogenesis of thoracic aortic disease, several feasible steps could improve the translational value of such models. Preclinical work could move beyond young, healthy, male animals toward aged and comorbid models of both sexes; distinguish prevention from treatment by intervening on already‐established disease rather than preventing its onset; resolve the aortic segment involved, so that the predominantly descending, type B phenotype of the BAPN model is not equated with the ascending and root disease of bicuspid aortopathy; and characterize the smooth muscle cell differentiation state, ideally through single‐cell or spatial profiling, so that protective mechanisms are interpreted within the context of the specific cellular substrate. Coupling such refined models with phenotypically stratified human tissue would place the promising findings of Zhao et al. on a firmer translational footing.

## Author Contributions

N. Grewal is the sole author and conceived, wrote, and approved the final manuscript.

## Funding

The author has nothing to report.

## Disclosure

The author has nothing to report.

## Conflicts of Interest

The author declares no conflicts of interest.

## Data Availability

The author has nothing to report.
